# Epidemiology of human RSV in Taiwan, 2018–2024

**DOI:** 10.1371/journal.pone.0357769

**Published:** 2026-09-17

**Authors:** Su-Lin Yang, Ming-Tsan Liu, Hsin-Hui Huang, Shu-Wei Lin, Yao-Tsun Li, Yu-Hsin Cheng, Fang-Tzy Wu

**Affiliations:** Center for Diagnostics and Vaccine Development, Centers for Disease Control, Ministry of Health and Welfare, Taipei, Taiwan; Chung Shan Medical University, TAIWAN

## Abstract

Respiratory syncytial virus (RSV) is a leading cause of lower respiratory tract infections in infants and older adults worldwide. This retrospective study investigated the epidemiology and molecular characteristics of RSV in Taiwan from 2018 to 2024 using data from the National Community Virus Surveillance System, which encompassed the coronavirus disease 2019 (COVID-19) pandemic period. Among 74,773 samples collected from patients with suspected respiratory viral infections, 1,224 samples tested positive for RSV, yielding an overall detection rate of 1.6%. RSV circulation was markedly disrupted during the COVID-19 pandemic, with no cases being detected during periods of stringent public health interventions and delayed outbreaks observed in late 2020 and 2023. Following the relaxation of nonpharmaceutical interventions, RSV seasonality shifted, with altered epidemic timing and changes in age distribution being observed. Most infections occurred in children younger than 5 years of age (92.6%), and RSV positivity varied significantly across age groups but not between sexes. Among the 1,224 isolates, 463 were randomly selected for glycoprotein (G) gene sequencing. The results revealed that RSV-A accounted for the majority (81.6%) of the tested isolates and were clustered within the A.D lineage, with A.D.3 being the dominant clade. The remaining strains were determined to be RSV-B, which were classified within the B.D lineage, with B.D.E.1 being observed as the dominant clade. The emergence of multiple RSV subclades during the study period indicates ongoing viral evolution in Taiwan. These findings demonstrate substantial shifts in RSV epidemiology following the COVID-19 pandemic and highlight viral evolution and clade replacement. Continued molecular surveillance is essential to monitor epidemiological trends and inform vaccination and prevention strategies.

## Introduction

Respiratory syncytial virus (RSV) was first identified in the United States in 1956 in infants with respiratory illness [1]. It is now recognized as a leading cause of lower respiratory tract infection (LRTI) worldwide. Globally, RSV is estimated to cause approximately 33 million LRTI cases on an annual basis among children younger than 5 years of age, thus resulting in 3.6 million hospitalizations and more than 118,000 deaths [2]. Although RSV infection is typically mild in healthy individuals, it can cause severe disease in infants, older adults, and immunocompromised patients; moreover, it most commonly presents as bronchiolitis or pneumonia [3–5]. In severe cases, progression to hypoxic respiratory failure may require intensive respiratory support, and extrapulmonary complications (including neurological involvement) have also been reported [6,7].

RSV belongs to the genus *Orthopneumovirus* within the family *Pneumoviridae*. It is an enveloped, single-stranded, negative-sense RNA virus with a genome of approximately 15.2 kb encoding 11 proteins [8]. Based on genetic variation in the surface glycoprotein (G) gene, RSV is classified into two major antigenic groups, including RSV-A and RSV-B, with each group comprising multiple genotypes. RSV-A genotypes include GA1–GA7, NA1, and ON1, whereas RSV-B genotypes include GB1–GB4, SAB1–SAB4, and BA1–BA10 [9–14]. The ON1 genotype of RSV-A and the BA genotype of RSV-B are characterized by nucleotide duplications within the G gene and have become globally predominant in recent years.

The association between RSV genotype and clinical severity remains unclear. Some studies have suggested that RSV-A infections are associated with more severe disease than RSV-B infections [15] and that specific genotypes (such as GA3) may be linked to increased clinical severity compared with other RSV-A genotypes [16]. In contrast, other studies have reported no significant association between RSV genotype and disease severity [17]. These discrepancies highlight the importance of ongoing molecular surveillance and comprehensive evaluation of clinical and virological data.

Since 1998, the Taiwan Centers for Disease Control (CDC) has established a central laboratory-based surveillance network to monitor trends in the circulation of respiratory viruses within the community [18]. Previous reports have indicated that RSV caused an unprecedented outbreak in 2020, with the incidence being increased 3.7-fold compared with that observed from 2010–2019, thus demonstrating this virus as the most common respiratory viral pathogen; moreover, this surge was associated with the emergence of the ON1 variant in 2020 [19]. The RSV-A ON1 genotype was first identified in 2011 and became the predominant circulating genotype after 2013, whereas the RSV-B BA9 genotype became predominant in 2012 in central Taiwan [20]. Subsequent sequencing analyses further demonstrated that all the isolated RSV-A strains belonged to the ON1 genotype and that all the RSV-B strains belonged to the BA9 genotype [21–23]. Phylogenetic analyses have also indicated that several novel RSV variants emerged during the coronavirus disease 2019 (COVID-19) pandemic in central and southern Taiwan [21–23]. However, these observations were limited to specific regions of Taiwan, and comprehensive nationwide data on the epidemiology and updated molecular characteristics of RSV are lacking. To address this research gap, we conducted a retrospective nationwide study to investigate the epidemiology, clinical characteristics, and molecular diversity of RSV in Taiwan from 2018 to 2024, thereby encompassing the COVID-19 pandemic period.

## Materials and methods

### Ethics statement

This study protocol was approved by the Institutional Review Board of the Taiwan Centers for Disease Control (IRB Nos. 107118, 109107, and 113106). The requirement for informed consent was waived by the Institutional Review Board.

### Human clinical samples

Nasopharyngeal or throat swab samples from patients with suspected respiratory viral infections were collected and submitted to contracted laboratories of the Taiwan Centers for Disease Control (CDC) for diagnostic testing and virus isolation between 1 January 2018 and 31 December 2024. All the samples were anonymized to protect patient confidentiality. Eight contracted laboratories located in northern, central, southern, and eastern Taiwan conducted community-based virus surveillance and contributed data on national epidemic trends. In total, 74,773 samples were tested during the study period, with approximately 9,744–11,687 samples being analyzed on an annual basis. Laboratory-confirmed RSV infections were identified via viral isolation in cell culture, and isolated RSV strains were subsequently forwarded to the Taiwan CDC for PCR-based genotyping and molecular analysis.

### Detection of co-detected respiratory pathogens

Clinical respiratory specimens were inoculated into MK-2, MDCK, HEp-2, HEL, Vero, and RD cell cultures. Viral isolates exhibiting cytopathic effects were identified using virus-specific immunofluorescence assays, including Light Diagnostics™ immunofluorescence assay kits (Millipore Corporation, Billerica, MA, USA) and the D³ Ultra 8™ DFA Respiratory Virus Screening and Identification Kit, D³ IFA Enterovirus Identification Kit, D³ DFA HSV Identification and Typing Kit, D³ DFA Metapneumovirus Identification Kit, and D³ DFA Cytomegalovirus Immediate Early Antigen Identification Kit (Diagnostic Hybrids, Athens, OH, USA). Co-detected respiratory viruses were identified by viral culture followed by immunofluorescence testing. *Mycoplasma pneumoniae* and SARS-CoV-2 co-detections were identified using the BioFire® Respiratory Panel 2.1 (bioMérieux, Marcy-l’Étoile, France), QIAstat-Dx® Respiratory Panel 2.0 (QIAGEN, Hilden, Germany), or LabTurbo Respiratory Multiplex Real-Time RT-PCR Kit (LabTurbo, New Taipei City, Taiwan), in accordance with the manufacturers’ instructions.

### Nucleic acid extraction

Nucleic acid extraction was performed using the TANBead Maelstrom 4800 automated extraction system (Taiwan Advanced Nanotech Inc., Taiwan) according to the manufacturer’s instructions, with 300 μL of each clinical sample being utilized. Sterile water was included as a negative control, and viral RNA extracted from a confirmed RSV strain was used as a positive control.

### Reverse transcription and PCR amplification

RSV genotyping was performed via reverse transcription PCR targeting the glycoprotein (G) gene, as previously described [24]. For RSV-A detection, the primers RSVA-5136F (5′-GTACCYTGCAGCATATGCA-3′) and RSVA-5679R (5′-CAAMTCCATTGTTATTTGCC-3′) were used to amplify a 544-bp fragment, whereas the primers RSVB-5114F (5′-ATGATTWYCAYTTTGAAGTGTTC-3′) and RSVB-5597R (5′-GAATAACTAAGCATRTGA-3′) were used to amplify a 484-bp fragment for RSV-B.

Reverse transcription was performed using SuperScript IV Reverse Transcriptase (Invitrogen). Briefly, 2.5 μL of extracted RNA was mixed with 1.5 μL of random primers (3 μg/μL; Invitrogen), 0.5 μL of dNTPs (10 mM; Invitrogen), and 10 μL of diethylpyrocarbonate (DEPC)-treated water. The mixture was heated at 97 °C for 5 min and cooled to 4 °C, after which 4 μL of 5 × RT buffer, 1 μL of 0.1 M DTT, and 0.5 μL of SuperScript IV enzyme were added to a final volume of 20 μL. Reverse transcription was performed at 55 °C for 10 min and terminated at 80 °C for 10 min. PCR amplification was performed using the Qiagen HotStarTaq Master Mix Kit (QIAGEN). Each 25 μL reaction contained 3 μL of cDNA, 12.5 μL of HotStarTaq Master Mix, 0.5 μL of each primer (10 μM), and 8.5 μL of DEPC-treated water. The following PCR cycling conditions were utilized: 95 °C for 15 min, followed by 40 cycles of 94 °C for 1 min, 55 °C for 1 min, and 72 °C for 1 min, with a final extension at 72 °C for 10 min. PCR products were visualized on a 1.5% agarose gel to confirm the presence of the expected 544-bp (RSV-A) or 484-bp (RSV-B) amplicons.

### Nucleotide sequencing

The PCR products were subsequently purified using a Zymoclean Gel DNA Recovery Kit (Zymo Research, Irvine, California, USA). Nucleotide sequences were determined with an automated DNA sequencing kit and an ABI Prism 3730XL DNA sequencer (Applied Biosystems, Foster City, CA, USA) according to the manufacturer’s protocols. Overlapping nucleotide sequences were combined for analysis and edited with the Sequencher software package (v5.4.6, Gene Codes Corporation, Ann Arbor, MI, USA). Nucleotide sequences were submitted to GenBank. The accession numbers were PX963774-PX963789, PZ121224-PZ121256, PZ128959-PZ129011, PZ135777-PZ135817, PZ147592-PZ147660, PZ169805-PZ169916, PZ170033-PZ170061, and PZ177951-PZ178060.

### Characterization of RSV-A and RSV-B sequences using Nextclade

The G gene sequences of RSV-A and RSV-B were analyzed using Nextclade (https://clades.nextstrain.org/) [25]. The aforementioned sequences were aligned to the reference genomes *h*RSV*/A/England/397/2017* (EPI_ISL_412866) and *h*RSV*/B/Australia/*VIC-RCH*056/2019* (EPI_ISL_1653999), respectively. Clade assignment was determined based on phylogenetic placement on a fixed reference tree according to established Nextstrain definitions.

### Statistical analysis

Statistical associations between prevalence and age and sex were evaluated using Pearson’s chi-square (χ²) test. A p value of <0.05 was considered statistically significant. Data analysis was performed using Microsoft Excel (Microsoft Corporation, Redmond, WA, USA) and IBM SPSS Statistics version 22.0 (IBM Corp., Armonk, NY, USA).

## Results

### Annual distribution of RSV cases

**Fig 1:**
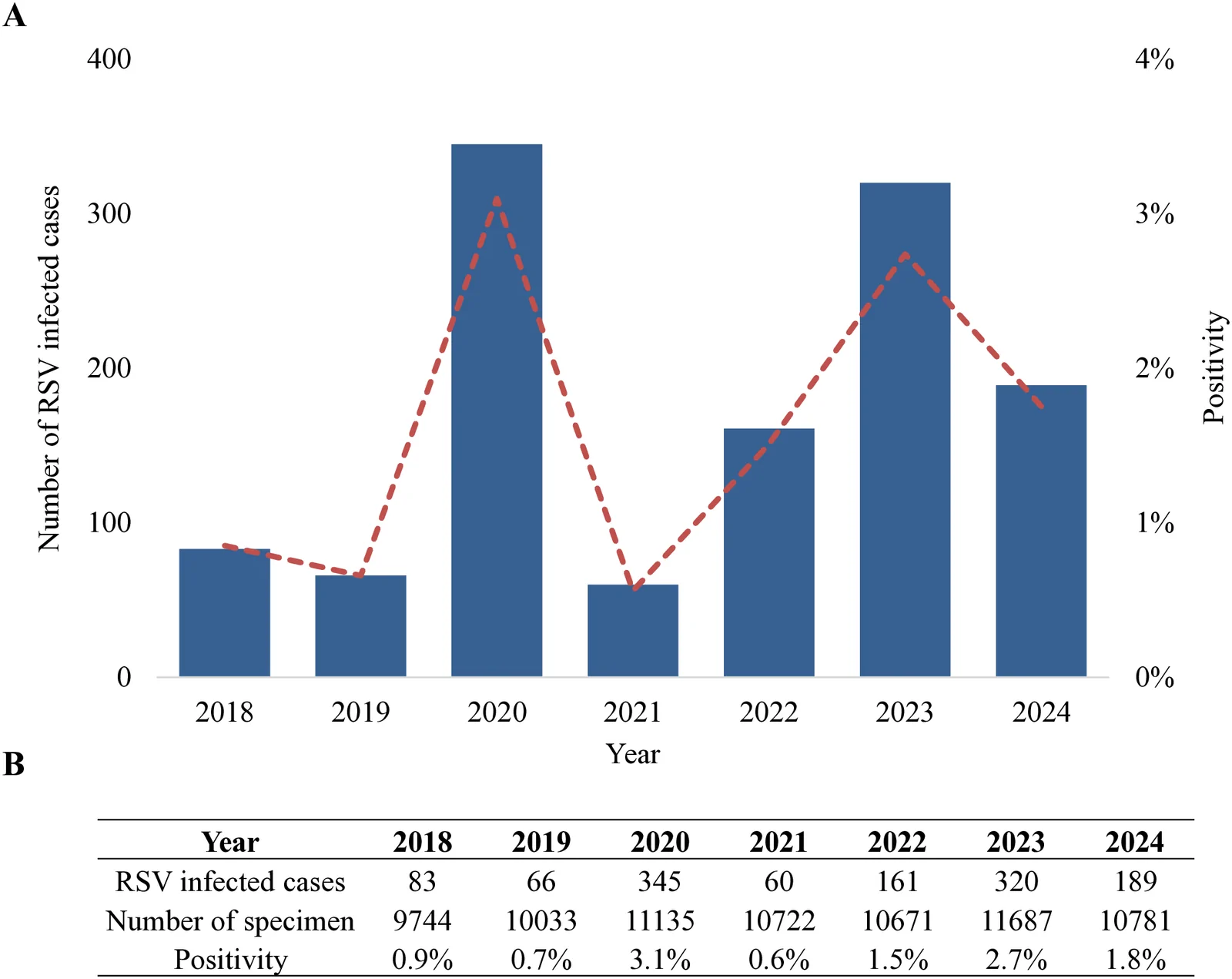
Annual community surveillance of RSV infection in Taiwan, 2018–2024. (A) Total number of tested samples and RSV detection rates. RSV was detected throughout the study period, with the highest detection rates being observed in 2020 and 2023 and the lowest rates being observed in 2021. (B) Annual number of RSV-positive samples and positivity rates. The overall positivity rate during the study period was 1.6%.

Between 2018 and 2024, a total of 74,773 samples were collected from patients with suspected respiratory viral infections, of which 1,224 tested positive for RSV, thereby corresponding to a detection rate of 1.6%. As shown in Fig 1, the annual number of RSV-positive cases ranged from approximately 60–345, with higher case numbers being observed in 2020 and 2023. The number of RSV infections in these years was approximately five- to sixfold greater than that observed in 2021.

### Monthly distribution of RSV cases

**Fig 2:**
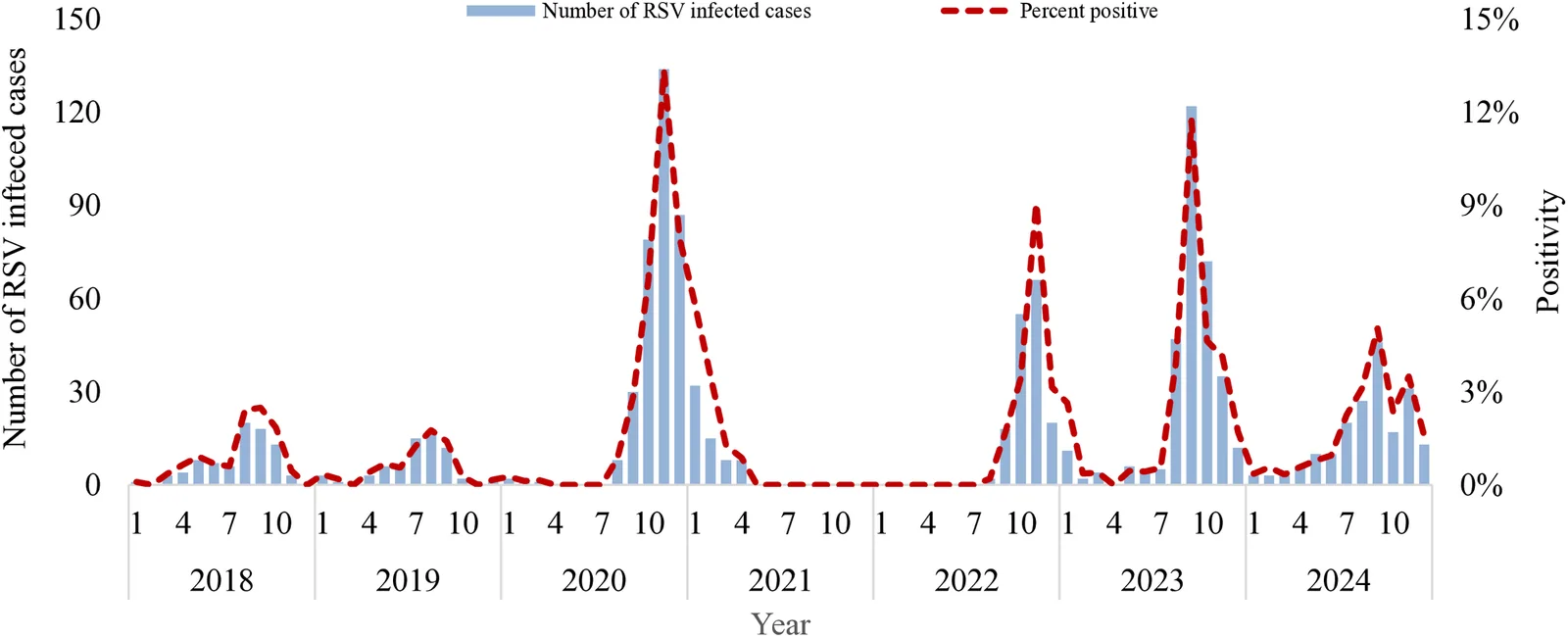
Monthly surveillance of RSV infections in Taiwan, 2018–2024. RSV activity predominantly occurred in the autumn during the 2018–2019 period. No RSV was detected between April and July 2020 or between May 2021 and July 2022. Increased RSV activity was observed in November 2020 and September 2023. In 2024, the seasonal pattern resembled that observed before the COVID-19 pandemic, with higher case numbers and positivity rates.

The monthly distribution of RSV infections from 2018 to 2024 is shown in Fig 2. Before the pandemic, RSV activity peaked in September in both 2018 and 2019. During the pandemic, no RSV cases were detected from April to July 2020 or from May 2021 to July 2022, and peak activity shifted to November in both 2020 and 2022. In the post-pandemic period, RSV activity again peaked in September in 2023 and 2024. The corresponding positivity rates for November 2020, November 2022, September 2023, and September 2024 were 13.4%, 9.1%, 11.7%, and 5.0%, respectively. The peak month therefore differed between the pandemic period and the pre-pandemic and post-pandemic periods.

### Distribution of RSV cases across cities and counties

The distribution of RSV cases and positivity rates across cities and counties was analyzed throughout the study period. During the prepandemic period (2018–2019), the highest numbers of RSV-positive cases were reported in Taichung City and Tainan City (S1 Fig panel A). During the pandemic period (2020–2022), Changhua County had the highest number of RSV-positive cases and the highest positivity rate (S1 Fig, panel B) This pattern continued to be observed in the postpandemic period (2023–2024), with Changhua County reporting the highest number of cases and positivity rates (S1 Fig, panel C).

RSV cases were not detected in Hsinchu County or Taitung County during the 2018–2019 period but were reported in both counties from 2020 to 2024. In contrast, no RSV cases were detected in Miaoli County or Yilan County during the 2020–2024 period. RSV infections were identified in Chiayi City and Chiayi County during the 2023–2024 period, whereas no cases were reported in these areas during the 2018–2022 period. Among the offshore islands, RSV was detected only in Penghu County in 2023.

### Sex distribution and age distribution of the RSV patients

**Fig 3:**
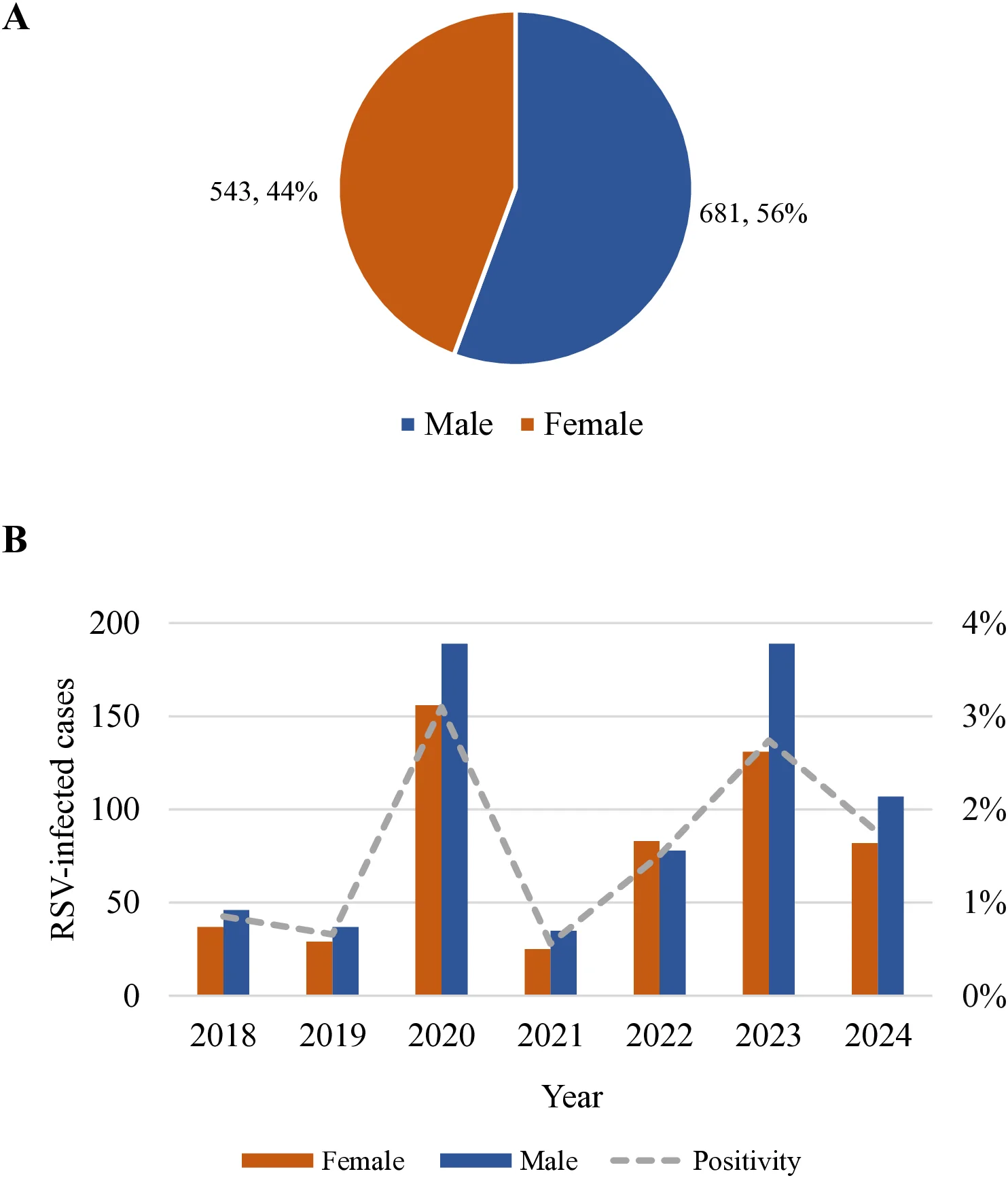
Sex distribution of RSV infections in Taiwan, 2018–2024. (A) Proportion of RSV infections by sex. Males accounted for a higher proportion of cases (56%) than females (44%). (B) Annual number of infections and positivity rates by sex; female cases slightly exceeded male cases in 2022.

**Fig 4:**
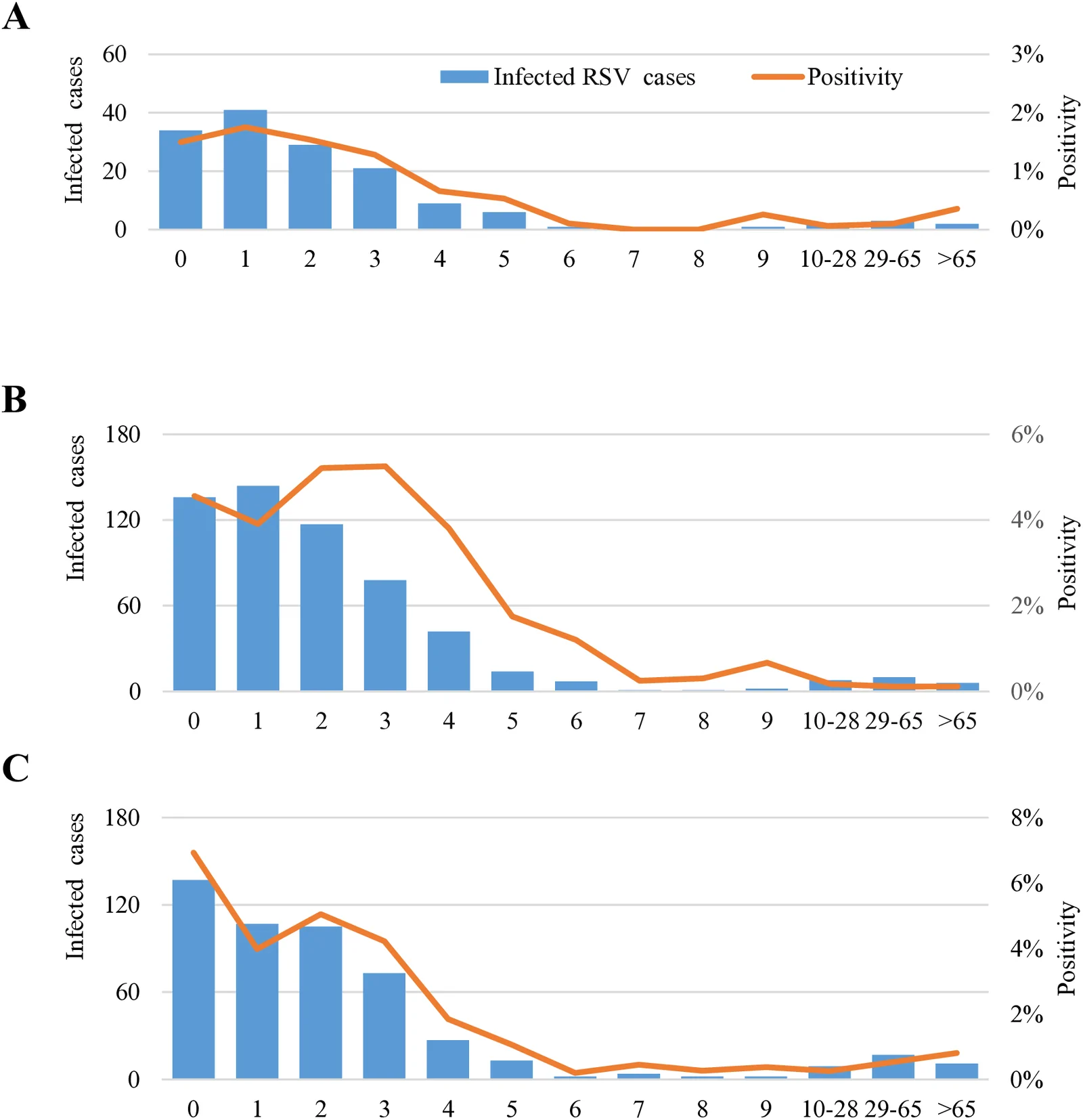
Age distribution and RSV positivity rates in Taiwan, 2018–2024. (A) Prepandemic period (2018–2019): most RSV cases occurred among children aged 1 year. (B) Pandemic period (2020–2022): the greatest number of cases was observed among children aged 2–3 years. (C) Postpandemic period (2023–2024): the greatest number of cases and positivity rates were observed among children younger than 1 year.

The sex distribution of RSV infections during the study period is shown in Fig 3. Males accounted for 56% of the reported cases. Although the number of female cases slightly exceeded that of male cases in 2022, no significant difference in sex distribution was observed across the study years from 2018 to 2024 (p = 0.512). The age distribution and RSV positivity rates among patients with suspected respiratory viral infections are shown in Fig 4. RSV positivity rates differed significantly among age groups, with children aged <5 years having significantly higher positivity rates than those in the other age groups (p = 0.021). The median age of the infected patients was 2 years (range, 0–99 years), with a mean age of 4.2 years. Before the COVID-19 pandemic (2018–2019), RSV detection rates were observed to be highest among children aged 1 year (Fig 4A). During the pandemic period (2020–2022), the highest detection rates were observed among children aged 2–3 years (Fig 4B). In the postpandemic period (2023–2024), the highest detection rates were observed among children younger than 1 year of age (Fig 4C). Among individuals aged ≥65 years, the detection rates were 0.4%, 0.1%, and 0.8% before, during, and after the pandemic, respectively.

### Sample sources and RSV positivity

**Fig 5:**
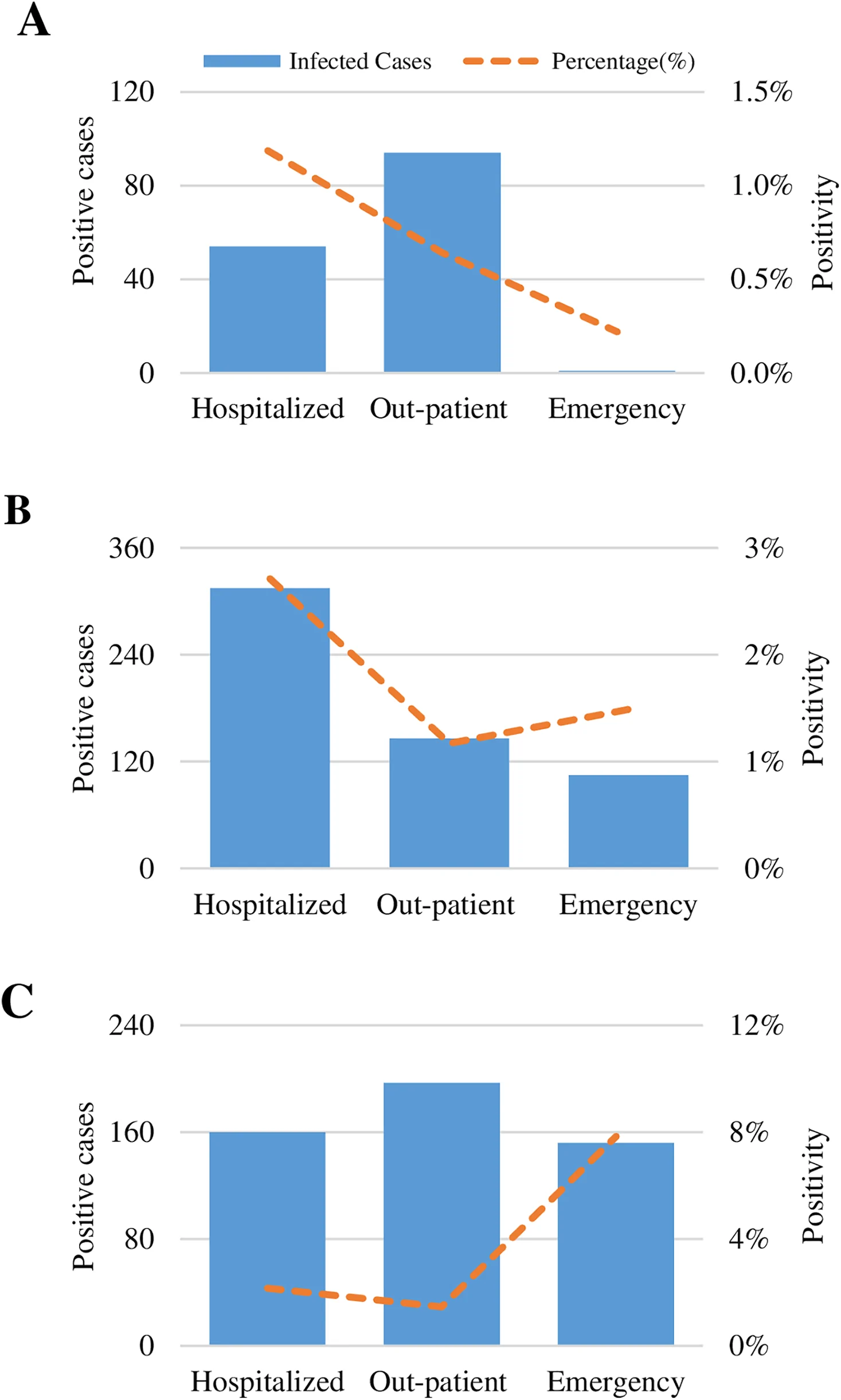
Sample sources and RSV positivity rates in Taiwan, 2018–2024. (A) Prepandemic period (2018–2019): most samples were collected from outpatients, whereas the highest positivity rates were observed among hospitalized patients. (B) Pandemic period (2020–2022): most samples were collected from hospitalized patients, who also demonstrated the highest positivity rates. (C) Postpandemic period (2023–2024): most samples were collected from outpatients, whereas the highest positivity rates were observed in emergency department patients.

The sources of the samples included in this study are shown in Fig 5. Before the COVID-19 pandemic (2018–2019), most of the samples were collected from outpatient settings (Fig 5A). During the pandemic period (2020–2022), the majority of the samples were obtained from hospitalized patients (Fig 5B); additionally, during the postpandemic period (2023–2024), most of the samples were collected from outpatients (Fig 5C). Before and during the pandemic, the highest RSV detection rates were observed among hospitalized patients. In contrast, during the postpandemic period, samples collected from emergency departments demonstrated the highest RSV positivity rate (7.8%).

### Clinical manifestations of RSV infection

Table 1 summarizes the clinical syndromes associated with RSV infection. Most of the patients presented with mild symptoms. Gastrointestinal manifestations, including vomiting and diarrhea, were reported in 4.4% and 1.8% of the patients, respectively. Syndromes commonly associated with enterovirus infection (such as herpangina and hand, foot, and mouth disease) were also observed. No severe complications (including pneumonia or central nervous system involvement) were identified during the study period.

**Table 1:** Clinical characteristics of RSV in Taiwan from 2018–2024.

Clinical syndrome	Total	Percentage
Fever	764	62.4%
Cough	498	40.7%
Rhinorrhea	305	24.9%
Sore throat	134	10.9%
Vomiting	54	4.4%
Malaise	35	2.9%
Headache	26	2.1%
Diarrhea	22	1.8%
Myalgia	21	1.7%
Herpangina	16	1.3%
Skin rash	15	1.2%
Pharyngeal vesicles or ulcers	10	0.8%
Acute upper respiratory infection	6	0.5%
HFMD	4	0.3%
Prostration	3	0.2%

### Codetection in RSV-positive patients

Table 2 summarizes the respiratory pathogens that were codetected in RSV-positive patients from 2018 to 2024. A total of 66 cases were codetected with other respiratory pathogens. Adenovirus was the most frequently codetected virus (37.9%, 25/66), followed by enterovirus and parainfluenza virus, each accounting for 19.7% (13/66). Among the parainfluenza virus codetections, type 3 was the predominant subtype. Codetections involving different enterovirus species were also identified, with coxsackievirus A being the most commonly detected species (16.7%, 11/66). No significant association was observed between RSV codetection and the severity of clinical syndromes.

**Table 2:** Codetection of RSV with other respiratory viruses in Taiwan, 2018–2024.

Codetected pathogens	No. of codetected pathogens	Percentage (%)
ADENO	25	37.9%
PARAINF Total	13	19.7%
PARAINF 3	(11)	
PARAINF 1	(1)	
PARAINF 4	(1)	
Enterovirus Total	13	19.7%
CA6	(3)	
CA10	(3)	
CA5	(2)	
CA2	(1)	
CA4	(1)	
CA16	(1)	
EVD-68	(1)	
ECHO4	(1)	
Influenza Total	5	7.6%
H3N2	(4)	
H1N1	(1)	
Metapneumovirus	4	6.1%
*Mycoplasma pneumoniae*	2	3.0%
CMV	2	3.0%
SARS-CoV-2	1	1.5%
HSV1	1	1.5%
Total	66	

Adeno, adenovirus; PARAINF, parainfluenza virus; CA, coxsackievirus; EV-D68, enterovirus D68; ECHO, echovirus; CMV, cytomegalovirus; HSV, herpes simplex virus.

### Nextclade and phylogenetic analysis of the RSV strains

During the study period, 1,224 RSV-positive isolates were identified, 463 of which were randomly selected for glycoprotein (G) gene sequencing. The numbers of sequenced isolates ranging from 2018 to 2024 were 23, 23, 113, 36, 72, 127, and 69, respectively. Among these isolates, 81.6% were classified as RSV-A, and 18.4% were classified as RSV-B. RSV-A was predominantly observed throughout the study period, and no RSV-B strains were detected in 2021.

**Fig 6:**
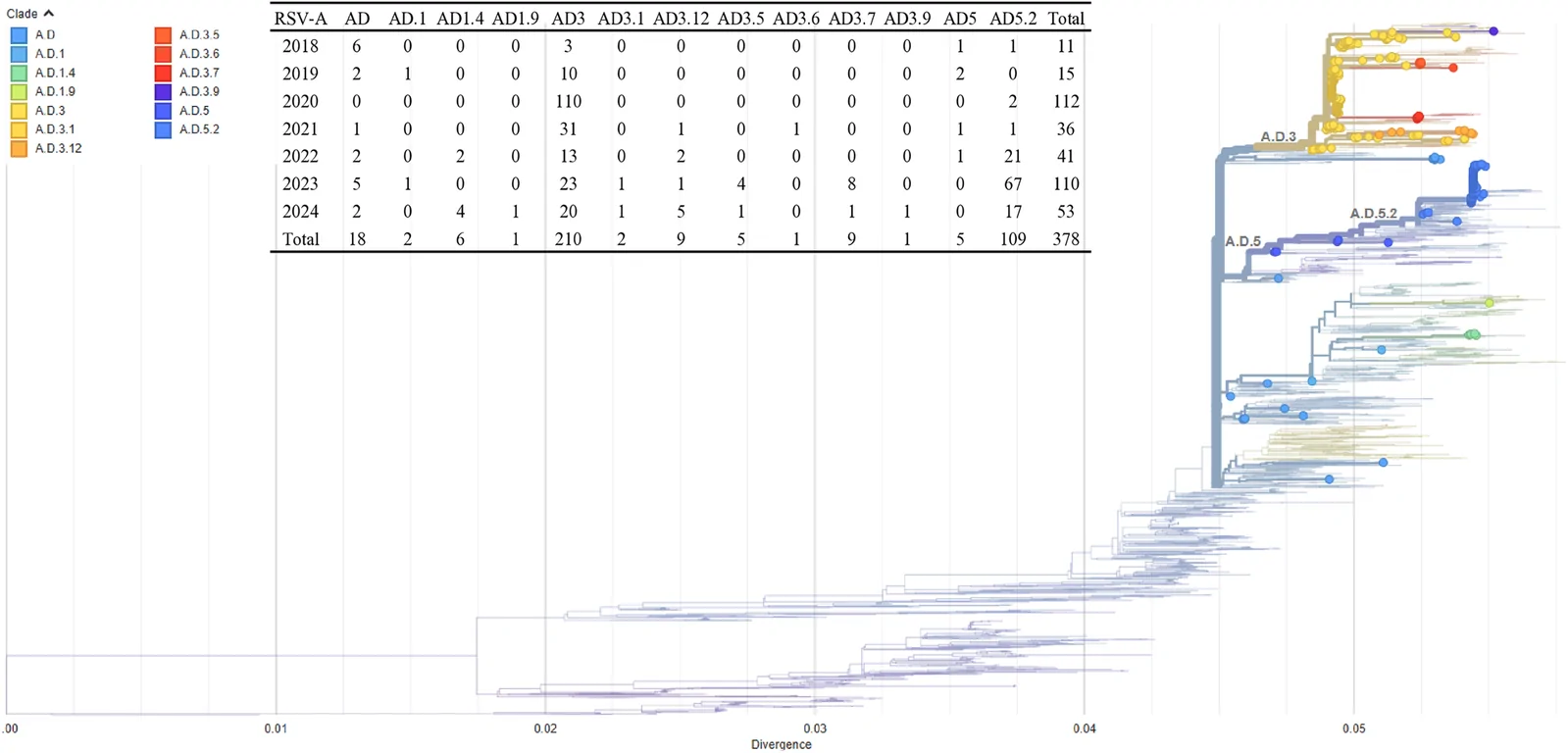
Nextclade analysis of RSV-A sequences in Taiwan, 2018–2024. RSV-A sequences were classified into 13 A.D clades. A.D.3 was the predominant clade, followed by A.D.5.2. Several clades (A.D.1.9, A.D.3.1, A.D.3.5, A.D.3.7, and A.D.3.9) were detected during the 2023–2024 period.

Sequencing analysis of the second hypervariable region of the G protein (amino acids 213–320) revealed that all the RSV-A strains were clustered within the A.D lineage. Nextclade analysis further classified the RSV-A sequences into 13 A.D clades (Fig 6). A.D.3 was the predominant clade, accounting for 55.6% (210/378) of the RSV-A strains, followed by A.D.5.2 (28.8%, 109/378). The A.D.3.6 and A.D.5 clades were not detected during the 2023–2024 period, whereas A.D.3.1, A.D.3.5, and A.D.3.7 were detected during this period. In addition, A.D.1.9 and A.D.3.9 were detected only in 2024.

**Fig 7:**
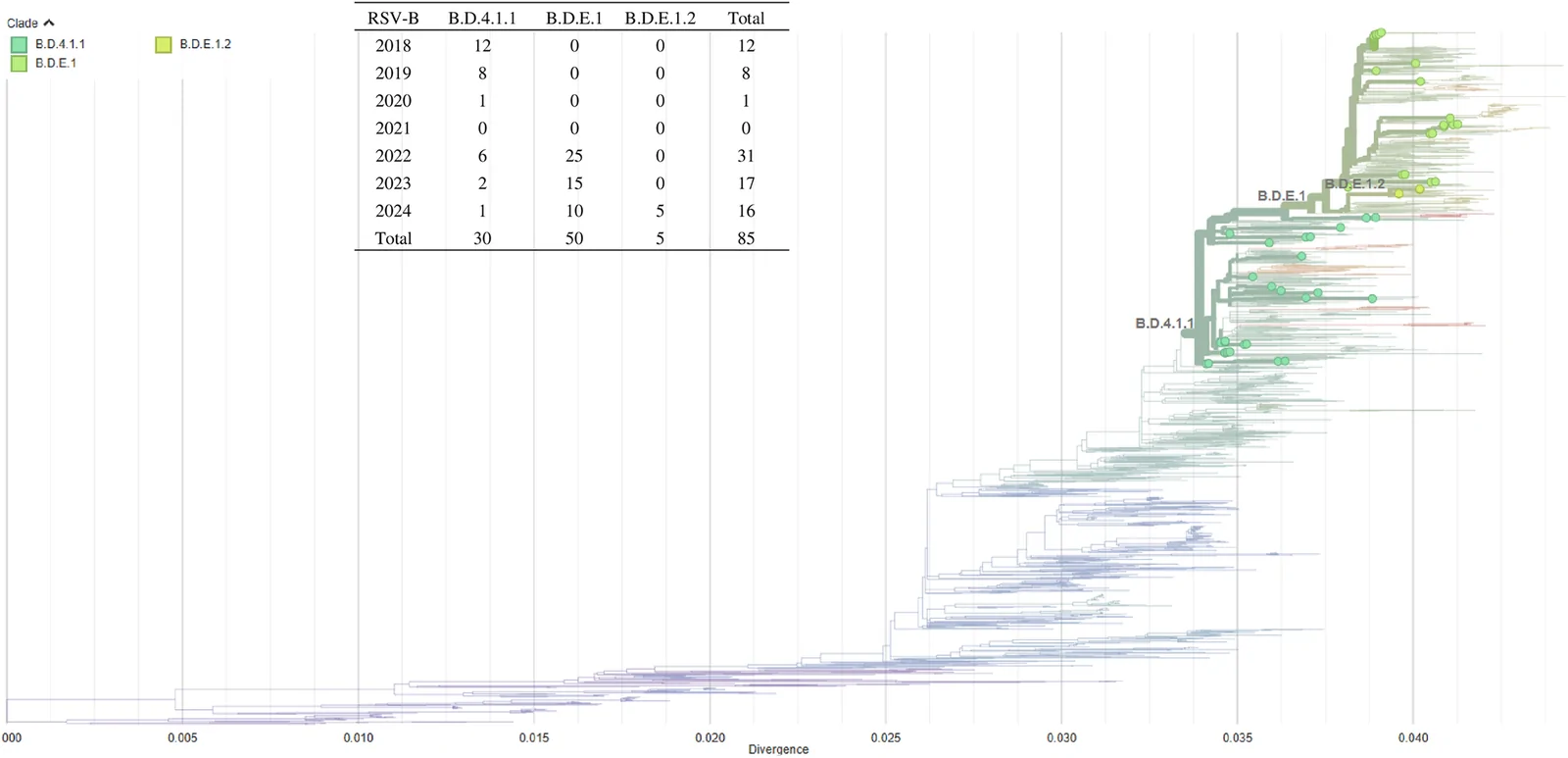
Nextclade analysis of RSV-B sequences in Taiwan, 2018–2024. RSV-B sequences were classified into three B.D clades. B.D.E.1 was the predominant clade during the study period. B.D.4.1.1 was predominant before 2020, whereas B.D.E.1 became predominant during 2022–2024.

With respect to RSV-B, all the strains were assigned to the B.D lineage. As shown in Fig 7, the RSV-B sequences were further classified into the following three clades: B.D.4.1.1, B.D.E.1, and B.D.E.1.2. B.D.E.1 was the predominant clade, accounting for 58.8% (50/85) of the strains, followed by B.D.4.1.1 (35.3%, 30/85). During the 2018–2020 period, all the RSV-B strains belonged to B.D.4.1.1. After 2020, the prevalence of B.D.4.1.1 declined, whereas B.D.E.1 became predominant. The subclade B.D.E.1.2 was detected in 2024.

## Discussion

RSV is a major pathogen that causes lower respiratory tract infections worldwide, with a particularly significant effect on infants and young children. This study retrospectively analyzed the epidemiology of RSV in Taiwan from 2018 to 2024, which encompassed the COVID-19 pandemic and the implementation of strict nonpharmaceutical interventions (NPIs). During this period, RSV activity was markedly disrupted, with no cases being detected at the peak of the pandemic, which likely reflects the impact of widespread NPIs. As these measures were gradually relaxed, delayed outbreaks were observed in 2020 and 2023, with RSV positivity rates increasing by approximately three- to fourfold compared with those during the preCOVID-19 period. Interestingly, the Irish study [26] reached conclusions broadly consistent with ours. Over comparable study periods, both studies identified young children as the most affected age group and observed marked shifts in RSV seasonality associated with COVID-19-related non-pharmaceutical interventions. Similar postpandemic shifts in RSV seasonality and increased outbreak intensity have been reported in several countries [27–34].

Before 2019, RSV circulated in Taiwan with a consistent seasonal pattern. However, the COVID-19 pandemic substantially altered the epidemiology of RSV, thus resulting in shifts in both its seasonal timing and age distribution. In this study, RSV activity peaked in September during both the pre-pandemic (2018–2019) and post-pandemic (2023–2024) periods. In contrast, during the pandemic, peak activity occurred later, in November 2020 and November 2022. The average detection rates during the pre-pandemic, pandemic, and post-pandemic periods were 2.0%, 11.3%, and 8.4%, respectively. These results indicate a temporary delay in epidemic timing and a shift in the seasonal pattern of RSV activity during the COVID-19 pandemic.

In this study, no significant difference in the sex distribution of RSV-positive cases was observed across the study years. In contrast, RSV positivity rates differed significantly among age groups, with children aged <5 years having higher positivity rates than those in the other age groups. The age distribution of RSV infections also changed over time. The highest detection rates were observed among children aged 1 year before the pandemic, shifted to children aged 2–3 years during the pandemic, and were highest among infants aged <1 year in the postpandemic period. Similar age-related changes have been reported in other countries [35–37], suggesting alterations in population susceptibility and immunity following the prolonged suppression of RSV circulation.

Sequencing analysis revealed that all the RSV-A strains were clustered within the A.D lineage, thereby corresponding to the ON1 genotype. Since its emergence in 2010, this genotype has become the predominant circulating RSV-A genotype worldwide, with the continued emergence of novel ON1 variants being reported [12,19,21–23,38–47]. In this study, A.D.3 was the predominant RSV-A clade and was detected throughout the study period, which is consistent with reports from other countries [32,48]. Temporal analysis revealed that the distribution of viral clades changed over time; specifically, A.D was predominant in 2018, followed by A.D.3 during the 2019–2021 period. A.D.5.2 replaced A.D.3 as the dominant lineage in 2022–2023, whereas A.D.3 again became predominant in 2024. In addition, other novel A.D subclades were detected during the postpandemic period.

During the 2018–2024 period, all the RSV-B strains were classified within the B.D lineage, thereby corresponding to the BA genotype, which is characterized by a 60-nucleotide duplication in the G gene [13]. Prior to 2020, B.D.4.1.1 was the predominant clade, and no RSV-B strains were detected in 2021. Subsequently, B.D.E.1 emerged and replaced B.D.4.1.1 as the predominant clade. The detection of B.D.E.1.2 in 2024 further indicates ongoing genetic evolution. These findings are consistent with global reports describing continued evolution and clade turnover within RSV-B lineages [46–50]. In this study, multiple A.D and B.D subclades were identified in Taiwan during the 2018–2024 period, thus highlighting substantial genetic diversity and ongoing viral evolution.

RSV frequently cooccurs with other respiratory viruses in clinical infections [51–57]. In this study, codetection of other respiratory pathogens was observed in 1.6% of the cases, which is lower than the rates reported in previous studies [56,57]. This difference may be attributable to the use of viral culture-based methods, which exhibit lower sensitivity than the multiplex real-time RT-PCR assays that are commonly used in other reports. In this study, no significant association was observed between RSV codetection and the severity of clinical syndromes.

This study has several limitations. First, the surveillance data may underestimate the true infection rate, as asymptomatic individuals and those who did not seek medical care were not included. In addition, RSV detection during the study period relied on viral culture rather than more sensitive PCR-based methods. Consequently, specimens with low viral loads or reduced viral viability may have remained undetected, potentially leading to an underestimation of RSV detection rates. Furthermore, the classification of clinical syndromes relied primarily on medical records and patient-reported symptoms, which may have introduced information bias. Second, the geographic distribution of detected cases may have been influenced by regional differences in healthcare-seeking behavior, testing intensity and laboratory practices, population density, healthcare resources, and the reporting coverage and locations of surveillance sites. These factors may have contributed to the higher number of RSV cases detected in Changhua County. Because they were not formally assessed, the observed geographic distribution may be subject to bias. Third, the phylogenetic analyses were based on partial G-gene sequences rather than whole-genome data, which limited the resolution of evolutionary relationships. Nevertheless, an Irish study demonstrated high concordance between lineage assignments based on G ectodomain sequences and those based on whole-genome sequences, supporting the utility of partial G-gene sequencing for RSV lineage classification [26] Finally, F gene sequencing was not performed, thus precluding the assessment of mutations associated with antiviral resistance or antibody escape.

In summary, the epidemiology of RSV in Taiwan was substantially altered during and after the COVID-19 pandemic, accompanied by ongoing viral evolution and changes in lineage distribution. Currently, three vaccines and monoclonal antibodies targeting the RSV prefusion F protein are available for the prevention of severe RSV infection in older adults, pregnant women, and infants [58–63]; however, antiviral treatment options remain limited. Continued surveillance to monitor epidemiological trends and circulating RSV strains, along with the development of effective immunoprophylaxis and improved therapeutic strategies, will be crucial in the future.

## Supporting information

S1 Fig Distribution of RSV cases and positivity rates across cities and counties in Taiwan, 2018–2024. (A) During the prepandemic period (2018–2019), the highest numbers of RSV-positive cases were reported in Taichung City and Tainan City. (B) During the pandemic period (2020–2022), Changhua County had the highest number of RSV-positive cases and the highest positivity rate. (C) During the postpandemic period (2023–2024), Changhua County continued to have the highest number of RSV-positive cases and the highest positivity rate.
(TIF)
